# Our New York Letter

**Published:** 1890-04

**Authors:** Wm. L. Russell

**Affiliations:** New York


### OUR NEW YORK LETTER.

NEW YORK, March 12, 1890.

The therapeutics of chronic heart disease was the subject before the Academy meeting of February 20th. The most important point referred to was the so-called mechanical treatment. Expressed in simple terms this means the employment of regulated systematic bodily exercise as a therapeutic measure for disease of the heart. The paper of the evening was read by Dr. H. N. Heineman, Professor of Practice at the Polyclinic. After enumerating the various morbid conditions and pathological changes to which the heart was subject; he stated that in them all the organ sought to resume its normal vigor. So long as it was successful in this there should be no treatment. Signs of failure, however, were indications for certain therapeutic measures. These could be classed as hygienic, dietetic, medicinal and mechanical. The hygienic measures were familiar to all. The medicinal treatment consisted in the use of heart stimulants and sedatives. Of the former digitalis was best, but its combination with convallaria rendered it more effectual; strophanthus and convallaria combined were also more powerful than either used singly. Strychnia and ergot given to their full physiological limit were valuable adjuvants.

Our knowledge of the value of dietetic and mechanical measures had been greatly developed by Oertel. The general dietetic rules to be followed were to increase all the food in old and weakly persons, and to decrease the fat-forming substances, and increase the albuminoids when there was an abnormal deposit of adipose tissue. Much could be done to relieve the work of the heart by attention to the quality and quantity of the food. Small quantities should be given at a time, and no fluid allowed with solid food. Fat absorption was promoted by abstention from fluids.

The mechanical treatment consisted of outdoor exercise, gymnastics and baths. Exercise improved the nutrition of all muscular tissue, and the heart was as amenable to this influence as other muscles. Well regulated mountain climbing, as prescribed by Oertel, was of the greatest benefit in certain cases. Massage and Swedish movements were preferred by some, but out-door exercise was unquestionably better. It was difficult to decide how long mechanical treatment should be continued. It was certain that it should be stopped if marked circulatory disturbance or increase of dilatation appeared.

Dr. Delafield, Professor of Practice at the College of Physicians and Surgeons believed that no plan of treatment was more worthy of consideration than the mechanical. When first suggested it shocked our preconceived notions concerning the treatment of heart disease. It had been well tried, however, and the results were important. The exercise should be suited to the heart action, not to the lesion. An improvement in the arterial circulation resulted, and the tendency to venous congestion was diminished. No cases were cured, however, and improvement for a moderate length of time was all that could be expected.

Dr. W. H. Thomson said that what muscle needed most was oxygen. This was illustrated in insects whose muscular power was enormous. This respiratory power was proportionately great; the whole surface of the body being engaged in this function. As much oxygen as possible should therefore be given to the heart muscle. Iron was the great oxygen carrier, and it was his custom to give it in all cases. Fresh air was more important than exercise. Camping out was a good plan. When there was much dilatation, swinging in a hammock or gentle strolling was all that should be allowed. An important point was the fact that most cases of heart disease occurring in adult and post-adult life were due to arterial changes. In such cases it was useless to increase the power of the heart. Life in the open air lowered the arterial tension and dilated the capillaries, and was thus of the greates t benefit, especally in cases cf fatty heart.

Dr. R. Van Santvoord stated that at the last German Congress for the study of Internal Medicine it was reported that many cases had done badly under the mechanical treatment. It would seem then that the contraindications had not been sufficiently well defined.

Dr. Loomis said that he always found it very difficult to decide whether to advise a patient with heart disease to exercise or to rest. He had seen two or three cases killed by ordinary exercise. It was a good plan to test the heart by having the patient walk about the room and respire vigorously. If the slightest waver was then discovered exercise should not be advised. In all cases past middle life very little exercise should be allowed. The seaside was better than a mountain climate. Cardiac stimulants were of only temporary benefit. When they were found to be necessary, a point had been reached where exercise could not be safely advised.

A very interesting discussion occurred at the last meeting of the Academy concerning the ultimate results of laparotomy for diseases of the uterine appendages. Dr. H. C. Coe read a paper. He said that a large number of women, many of whom had been operated on by the most experienced laparotomists in New York, had subsequently come under his observation at the dispensary to which they had applied for further relief. His observations had led him to believe that permanent ill effects followed the operation in a great many instances. He had done several secondary operations, and had made many autopsies, and had thus been able to ascertain the exact conditions.

The ill effects were of two varieties psychical and physical. Among the former were insanity and unsexing of the woman. The latter were persistent pelvic pain, vesical irritability, and intestinal obstruction. These were due to adhesions and to induration about the stump. So far as he knew there was no way of avoiding these conditions.

He then related short histories of ten cases. In all of these intra-pelvic pain continued after the operation, and in some it was more severe than before. In one a secondary operation revealed a mass of adhesions which could not be removed, and which obliterated the ureter, causing pyonephrosis and death. In a second there was found an ulcer of the intestine caused by the pressure of an indurated mass around the stump. In other cases a tender indurated mass could be plainly felt at the site of the stump. These were only a few of many such cases which he had observed, and he therefore believed that many patients who were classed as "recovered" after laparotomy were by no means cured.

Dr. W. Gill Wylie remarked that the results of laparotomy for diseases of the uterine appendages were generally good; so good that the operation would be done more frequently in the future than it is at present. If there were complications, or if the nervous system were seriously affected, it was a different matter, and the results were not so good. In his experience the operation was a failure when done for the relief of what were thought to be reflex nervous disorders.

One of the principle causes of after troubles was failure in removing every particle of diseased tissue. If any were allowed to remain, cysts formed beneath the scar tissue and caused pain. This happened probably as often as once in every four or five operations. Sometimes a small portion of pyogenic tissue was left behind, or the ligature of the pedicle was septic, and, acting as a foreign body, produced an indurated mass which caused trouble.

Insanity was not so common as sometimes stated. When it occurred it was in cases in which there had been serious nerve trouble before the operation.

The effect on a woman was that of the menipause. If the operation were done on a girl before the age of puberty she might loose her womanly characteristics.

Adhesions caused no trouble unless diseased tissues were also present. Indeed he had seen adhesions, which completely filled the pelvis, disappear as if by magic in six months after the diseased tubes and ovaries had been removed.

Dr. C. C. Lee of the Woman's Hospital had classed as recovered ninety-nine out of a hundred and three cases in which he had done laparotomy for diseased tubes and ovaries. In following his cases for two years after the operation he had found that the result had been entirely satisfactory when the operation had been done for structural diseases which could be clearly made out beforehand. In cases where it could not be so clearly made out the result had been fairly good, while in those cases in which the operation had been done for neuroses such as epilepsy, hystero-epilepsy, pure hysteria and neuralgia no good had been accomplished. Not a single one of these cases was cured, and improvement was fitful and uncertain. He thought then that when it was clear that structural disease was present, the operation offered the only chance of recovery.

Dr. H. C. Hanks believed that when pyo-salpinx was present the operation should always be done. He had seen no good result when it was performed simply for the relief of neurotic conditions.

Dr. A. P. Dudley had noticed that in all his cases in which pelvic pain had recurred silk had been used to ligate the pedicle. He now used cat-gut large enough to require four days for its absorption. He believed that the custom of keeping the bowels locked was a prominent cause of adhesions. For this reason he now had them moved very soon after the operation, sometimes giving a saline as early as an hour after the anæsthetic.

Dr. Wm. T. Lusk regarded laparotomy as one of the most valuable contributions to uterine therapeutics. Many women had been raised from their beds by it, and many had been saved from death. The operations should always be done when it was positive that there was pus in the tubes. All cases of tender and dilated tubes, however, did not require the operation. He had seen two such cases recently in which simple measures had produced an entire cure. He was not altogether satisfied with the results of laparotomy. He had seen a number of cases of melancholia resulting from it. He believed then that it should not be resorted to until a prolonged trial of other measures had proved fruitless.

Dr. Coe, in closing, spoke of the great importance attached to the more or less permanent psychical effects which resulted in many instances.

WM. L. RUSSELL.

